# Bioconversion of α-Linolenic Acid into n-3 Long-Chain Polyunsaturated Fatty Acid in Hepatocytes and *Ad Hoc* Cell Culture Optimisation

**DOI:** 10.1371/journal.pone.0073719

**Published:** 2013-09-11

**Authors:** Ramez Alhazzaa, Andrew J. Sinclair, Giovanni M. Turchini

**Affiliations:** 1 School of Life and Environmental Sciences, Deakin University, Victoria, Australia; 2 School of Medicine, Deakin University, Victoria, Australia; National Institute of Nutrition, India

## Abstract

This study aimed to establish optimal conditions for a cell culture system that would allow the measurement of 18∶3n-3 (ALA) bioconversion into n-3 long-chain polyunsaturated fatty acid (n-3 LC-PUFA), and to determine the overall pathway kinetics. Using rat hepatocytes (FaO) as model cells, it was established that a maximum 20∶5n-3 (EPA) production from 50 µM ALA initial concentration was achieved after 3 days of incubation. Next, it was established that a gradual increase in the ALA concentration from 0 up to 125µM lead to a proportional increase in EPA, without concomitant increase in further elongated or desaturated products, such as 22∶5n-3 (DPA) and 22∶6n-3 (DHA) in 3 day incubations. Of interest, ALA bioconversion products were observed in the culture medium. Therefore, *in vitro* experiments disregarding the medium fatty acid content are underestimating the metabolism efficiency. The novel application of the fatty acid mass balance (FAMB) method on cell culture system (cells with medium) enabled quantifying the apparent enzymatic activities for the biosynthesis of n-3 LC-PUFA. The activity of the key enzymes was estimated and showed that, under these conditions, 50% (Km) of the theoretical maximal (V_max_ = 3654 µmol.g^−1^ of cell protein.hour^−1^) Fads2 activity on ALA can be achieved with 81 µM initial ALA. Interestingly, the apparent activity of Elovl2 (20∶5n-3 elongation) was the slowest amongst other biosynthesis steps. Therefore, the possible improvement of Elovl2 activity is suggested toward a more efficient DHA production from ALA. The present study proposed and described an *ad hoc* optimised cell culture conditions and methodology towards achieving a reliable experimental platform, using FAMB, to assist in studying the efficiency of ALA bioconversion into n-3 LC-PUFA in vitro. The FAMB proved to be a powerful and inexpensive method to generate a detailed description of the kinetics of n-3 LC-PUFA biosynthesis enzymes activities *in vitro*.

## Introduction

Polyunsaturated fatty acids (PUFA) are essential dietary nutrients for vertebrates [1], [2] and required for optimal health and normal development [3], [4]. There has been considerable progress in revealing the details of n-3 long-chain PUFA (LC-PUFA) biosynthesis and homeostasis *in vitro* and *in vivo*, benefiting from new analytical methods and approaches [5]–[7]. However, quantifying the endogenous conversion of 18∶3n-3 (ALA) into n-3 long-chain PUFA (LC-PUFA) is not yet optimised and still surrounded with confusion [8]–[11]. Species-specific, tissue-specific and other intrinsic factors appear to affect this bioconversion as physiological state and pathological conditions in vertebrates [12]–[16].

Liver is known to be the major organ for lipid metabolism [17], [18], where hepatocytes contain the necessary enzymes for the elongation and desaturation of ALA to 20∶5n-3 (EPA) and 22∶6n-3 (DHA) [19]–[21]. Therefore, hepatocytes have been used regularly in PUFA metabolism studies [22]–[24]. Numerous reports have inferred the kinetics of fatty acid (FA) metabolism by analysing FA composition in tissues after controlled feeding experiments, while other studies used *in vivo*, *ex vivo* or *in vitro* approaches with labelled FA [5], [6], [11], [25]. However, these methods can be expensive, not available for every laboratory, and their outputs differ widely depending on the analytical application and tissue(s) studied. A whole-body *in vivo* FA mass-balance (FAMB) method has enabled the estimation of the overall capacity of an organism to metabolise FA [26], [27]. It is envisaged that combining the advantage of results reproducibility obtained from a cell line platform with the FAMB approach could provide a detailed insight on the efficiency of ALA bioconversion into EPA and DHA.

In the present study, mammalian hepatocytes were utilised in order to establish an optimised experimental platform for investigating ALA conversion into n-3 LC-PUFA *in vitro*. The objectives were to determine the most effective duration and concentration of ALA to be converted into n-3 LC-PUFA in hepatocytes and to estimate the apparent enzymatic activities through this pathway by implementing FAMB on the whole flask. The current study is a contribution towards establishing a novel approach, a robust experimental platform and methodology for future nutrition biochemistry research and advancing the current knowledge on the efficiency of n-3 LC-PUFA metabolism in liver.

## Materials and Methods

### Cell Culture

Rat hepatoma cell line (FaO) was obtained from American Type Culture Collection (Bethesda, MD, USA) and grown in humidified atmosphere (95% air, 5% CO_2_) at 37°C in RPMI-1640 medium (GIBCO®, UK) supplemented with 10% (vol/vol) fetal bovine serum (SAFC Biosciences, KS, USA). Phosphate buffered saline, 1X (PBS) (Sigma-Aldrich, MO, USA) was used to wash the cells and a 0.25% trypsin-EDTA (GIBCO®, UK) was used for 3 min at 37°C to detach the cells from the flask.

### Experimental Design

**Figure 1:**
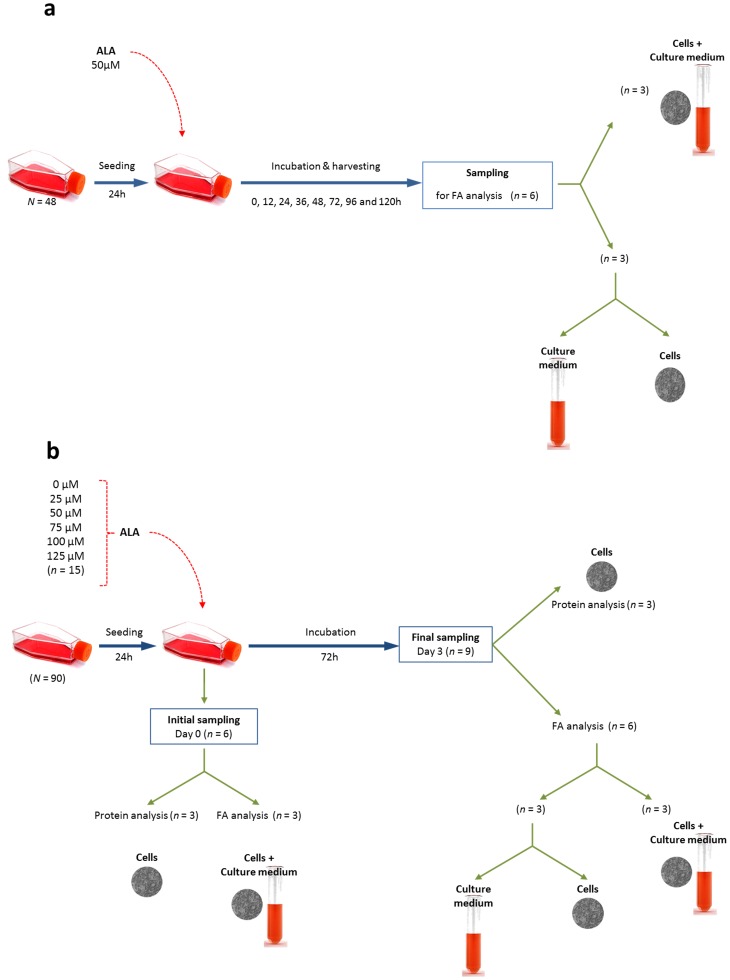
Experimental design for a: the effect of time of incubation, and b: effect of ALA substrate concentration. Experimental design for the flasks used in FAMB computation is also explained in the subfigure b.

Effect of time of incubation: cells were seeded in 48 small flasks (25 cm^2^) at initial density of 4–5 × 10^6^ cells under the conditions mentioned above. After 24 h of seeding, 50 µM of ALA was added to each flask. Samples of cells, the culture medium and the cells with their culture medium were harvested at 0, 12, 24, 36, 48, 72, 96 and 120 h post incubation with ALA (Figure 1a). Each sampling was conducted by aspirating the culture medium from six flasks into six separate tubes, the cells were then dislodged, harvested and pelleted at 300 *g* then washed twice. Three samples of washed cells were kept for further analysis while three other washed cell samples were mixed with their respective culture medium. Therefore, for each time point, three samples were collected from the cells alone, from the culture medium and from the combined cells and their culture medium (Figure 1a). All samples were kept at −20°C for further lipid extraction.

Effect of ALA substrate concentration: following the readings from the time of incubation, an incubation time of 72 h was selected. Cells were seeded at initial density of 4–5 × 10^6^ cells in 90 small flasks (25 cm^2^) for 24 h, then incubated with increasing concentrations of ALA (0, 25, 50, 75, 100 and 125 µM) (Figure 1b). Samples of cells were harvested and washed at time zero (initial harvest) and after 72 h (final harvest) to analyse FA composition and the protein content in the cells (Figure 1b). The FA composition was assessed on samples of cells, the culture medium and the cells with their culture medium, which were collected, processed and stored as mentioned above.

### Protein Content

Cells samples incubated with different concentrations of ALA (0, 25, 50, 75, 100 and 125 µM) were harvested at 0 and 72 h (Figure 1b), washed twice and their lysate content of total protein was quantified by BCA protein assay kit (Pierce, IL, USA).

### Lipid Extraction and FA Analysis

Samples of cells, the culture medium and the combination of cells and their culture medium were thawed and their total lipid were extracted in chloroform:methanol (2∶1) solvent overnight [28]. Following the lipid extraction, lipid classes from representative samples of the cells, medium and combination of the cells and the medium (n = 3, *N* = 9) were analysed from flasks supplemented with 50 µM of ALA for 3 days by thin layer chromatography (TLC) plates as described previously [29] using TLC 18-5 (Nu-Check Prep, Inc., MN, USA) as an external standard. For all samples, FA from total extracted lipid were esterified into methyl esters by acid-catalyzed methylation [30], [31]. Known concentrations of three internal standards (Sigma-Aldrich Inc., MO, USA) were included in each sample to monitor the accuracy of the lipid extraction, methylation and quantification as the following: 19∶0 was added before extracting the total lipid, 23∶0 was added before FA methylation, and 17∶0 methyl ester was added to each sample before injecting into the GC. FA methyl esters were isolated and identified using an Agilent 7890A GC (Agilent Technologies, USA) equipped with a DB-23 capillary column (60 m, 0.25 mm internal diameter, 0.15µm film thickness; Agilent) and a flame ionisation detector (FID). Samples (2µL each) were injected in a split mode (10∶1 ratio) by an Agilent 7693 autosampler injector and carried by Helium gas. After injection, the oven temperature was raised from 50°C at 8°C/min to 180°C, increased by 2°C.min^−1^ to 220°C then increased by 25°C.min^−1^ to 240°C and held for 4.95 min. Acquired peaks were quantified with Agilent Technologies ChemStation software, corrected by the theoretical relative FID response factors and, after confirmation of reliable lipid extraction and FA methylation implementation by comparison of the three internal standards used, individual FA were eventually quantified relative to the internal standard (23∶0).

### FA Metabolism Estimation

The estimation of the apparent FA metabolism (FA *de novo* production, β-oxidation, elongation and desaturation) was calculated by implementing the FAMB method as described earlier [7], [26], [32], with the following modifications: 1) the cell culture flask including the cells and their culture medium was considered as an independent entity (equivalent of the individual animal whole body in the original method), and individual FA quantities in whole flask (cells plus culture medium) were assessed; 2) three flasks for each concentration treatment were seeded with similar initial cell density and incubated for 24 h under the conditions mentioned above, then ALA was supplemented (0, 25, 50, 75, 100 and 125 µM) and the cells combined with their culture medium were immediately harvested and frozen until subsequent total FA analysis (initial flask FA content) (Figure 1b); 3) three flasks for each concentration treatment were seeded with similar initial cell density and different ALA concentration and cultured under the conditions mentioned above for 72 h, then harvested and kept frozen until subsequent FA analysis (final flask FA content) (Figure 1b); 4) six additional flasks for each concentration treatment where prepared and harvested as described at point 2 (three flasks) and point 3 (three flasks), and then used for protein quantification from the cells (Figure 1b); 5) the appearance/disappearance of individual FA was computed by difference between final flask FA content and the initial flask FA content. Data relative to individual FA appearance/disappearance were then converted from mg of FA per flask during the 72 h, into µmol g^−1^ of cellular protein hour^−1^. The subsequent backward computations along the known FA metabolic pathways were then implemented as described in the original method [26]. The availability of specific FA was computed in mmol.g^−1^ of cell protein by summing the initial concentration of the FA and the *de novo* production of it in the flask during incubation. The recorded apparent *in vitro* enzymatic activities were eventually reported as µmol.g^−1^ of cell protein.hour^−1^.

### Statistical Analysis

Data are presented as mean ± SE. The percentage data were arcsine transformed into angular degrees prior to analysis. One-way ANOVA tested the differences between groups and, when significant, was followed by Tukey’s post hoc test. All data were also analysed by linear regression relative to ALA concentration or the time after ALA supplementation and, when appropriate, further nonlinear regression trends were computed with Michaelis-Menten enzyme kinetics model, followed by D’Agostino & Pearson omnibus K2 test for normality of residual. Analyses were performed with SPSS ver. 20 (IBM, USA) and Prism ver. 5 (GraphPad Software Inc., USA).

## Results

### Effect of Time of Incubation

**Figure 2:**
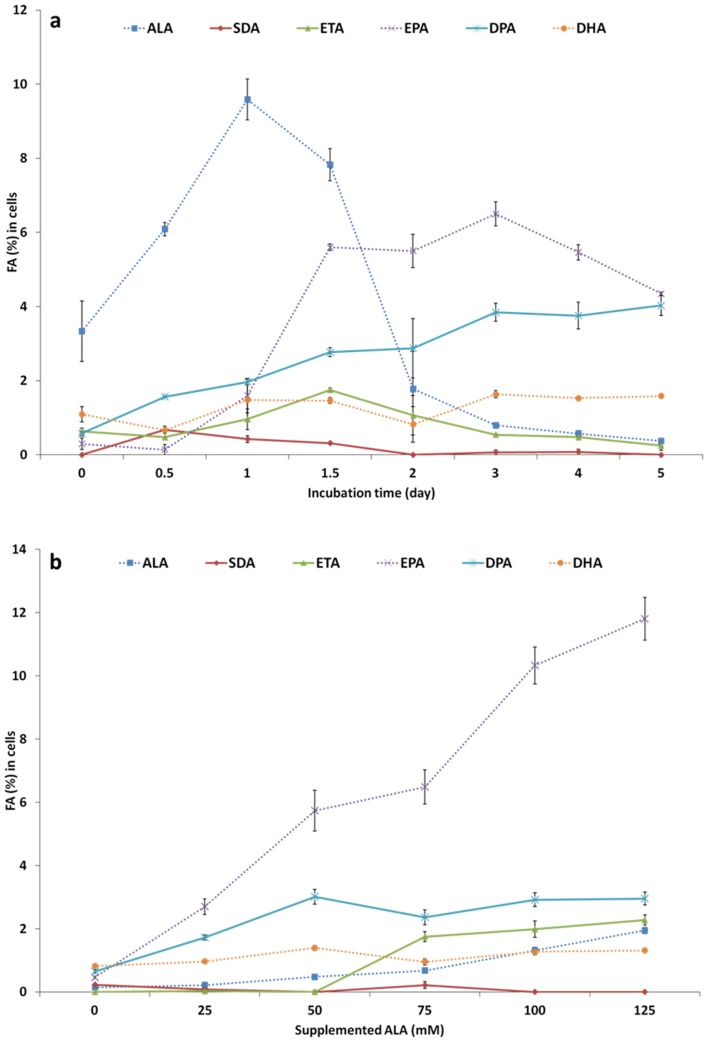
18∶3n-3 (ALA) bioconversion in FaO hepatocytes at: a; different time-points, b; at 3 days with different concentrations added into the culture medium (0, 25, 50, 75, 100 and 125 µM). SDA: 18∶4n-3, ETA: 20∶4n-3, EPA: 20∶5n-3, DPA: 22∶5n-3, DHA: 22∶6n-3.

Cells content of ALA increased and peaked at day 1, thereafter the concentration returned to almost zero by day 5 (Figure 2a, Table S1). The EPA proportion increased rapidly till day 3 and then decreased during the following two days. DPA (22∶5n-3) proportion rose slowly but steadily and by day 5 was present almost at the same level as EPA. EPA and DPA were the main n-3 FA from days 2 to 5 of the incubation. The proportion of DHA was remarkably lower than that of EPA and DPA, slightly increasing from day 0 to day 3, and then plateaued.

The culture medium had decreasing content of ALA and increasing EPA with the time (Table S2). A small, but significant proportion of 20∶3n-3 (ETrA) appeared in the medium at day 2. The ALA content in the whole flask (combined cells and the culture medium), decreased significantly with the time and corresponded with an increase in the EPA content, but not with DPA or DHA (Table S3).

### Effect of ALA Substrate Concentration

Increasing the ALA concentration, at 25 mM increments from 0 up to 125 mM, in cell incubated over a 3 days period, corresponded with a significant proportional increase in EPA content in the cells upto approximately 12% of total FA (Figure 2b, Table S4). The DPA, ETA (20∶4n-3) and DHA proportion increased significantly with ALA concentration, up to 3 and 1.3% of total FA, respectively. SDA (18∶4n-3) was always detected at extremely low levels for all ALA concentrations tested.

**Figure 3:**
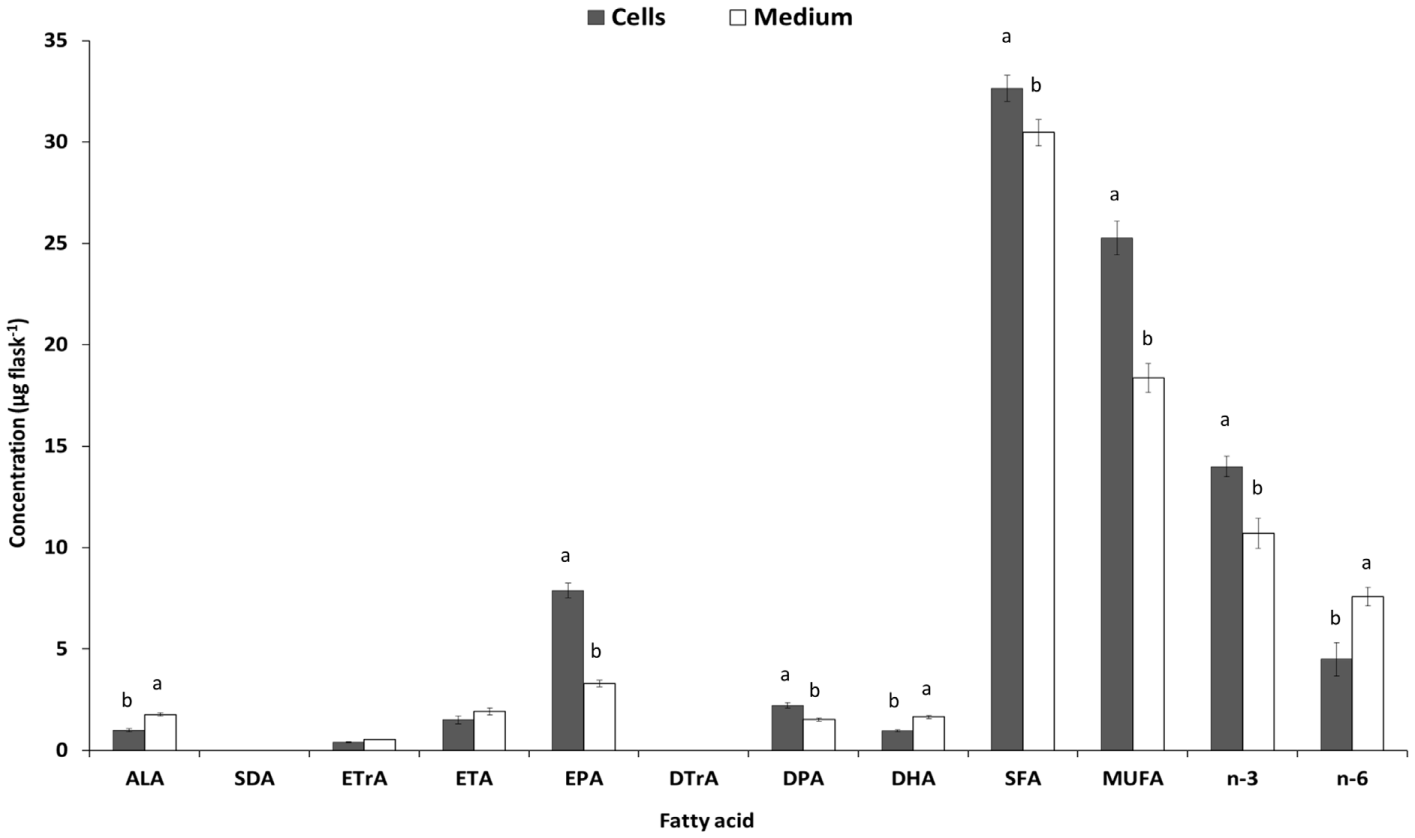
The concentration (µg flask^−1^) of different fatty acid and fatty acid groups in the cells or the culture medium of FaO after 3 days of supplementation with 100µM of ALA. Different letters above bars from the same fatty acid indicate significant (*P*<0.05) differences.

Similar changes were observed in the culture medium FA composition (Table S5), while the FA composition of the whole flask had similar patterns of changes for the n-3 LC-PUFA (Table S6). There was significantly greater concentration of EPA in the cells while more DHA was observed in the medium at different levels of ALA supplementation (presented for cells supplemented with 100µM ALA in Figure 3). Other products of ALA conversion, such as ETrA, ETA, EPA and DPA were also present in the culture medium. The proportion of n-3 PUFA out of the total FA in the cells was comparable to that in the whole flask (Tables S4 and 6). The proportion of n-3 PUFA out of the total FA in the culture medium was between 0.8 and 0.9 compared with the whole flask (Tables S5 and S6).

### Lipid Class

Most of the observed FA in the cells, their culture medium and in the whole flask were in the phospholipid fraction (80–85% of total lipid) and in the neutral lipid fraction as non-esterified FA (10–15% of total lipid). The main ALA bioconversion products in the phospholipid for the cells and the medium were EPA and DPA (9–13% of total phospholipid FA), with DHA and ETrA being next highest in proportion (Table 1). In the non-esterified FA fraction, the main ALA bioconversion products were ETrA and 22∶3n-3 (DTrA) (57–59% of total non-esterified FA), while EPA, DPA and DHA accounted for 22% of total non-esterified FA (Table 1).

**Table 1:** FA changes in the phospholipid and the non-esterified fatty acid classes, fractionated by TLC, in FaO cells, culture medium and the cells with their culture medium after 3 days of incubation.

FA %	Phospholipid				Non-esterified fatty acid							
	Cell	Medium	Cells+medium		Cell		Medium			Cells+medium		
**12∶0**	0.0±0.0	0.0±0.0		0.0±0.0		0.0±0.0		0.0±0.0			0.0±0.0	
**14∶0**	0.3±0.1	0.3±0.2		0.4±0.1		0.2±0.0		0.3±0.1			0.1±0.1	
**16∶0**	16.7±1.5a	19.7±0.7a		23.7±3.5a		6.7±0.2b		5.9±1.5b			5.9±2.6b	
**18∶0**	20.7±1.4a	21.9±1.1a		0.4±0.1b		7.0±0.3b		7.9±0.4b			7.4±0.8a	
**20∶0**	0.5±0.0	0.7±0.1a		0.8±0.1a		7.0±0.2		0.0±0.0b			0.0±0.0b	
**22∶0**	0.8±0.0a	1.8±0.0a		1.9±0.1a		0.0±0.0b		0.0±0.0b			0.0±0.0b	
**14∶1n-5**	0.1±0.1	0.2±0.1		0.0±0.0		0.4±0.2			0.1±0.1		0.0±0.0	
**16∶1n-7**	6.3±0.1a	3.5±0.2a		5.7±1.2a		0.5±0.3b		0.1±0.1b			0.3±0.1b	
**18∶1n-7**	5.3±0.1a	5.3±0.1a		6.8±0.4a		0.5±0.1		0.1±0.1b			1.9±0.1	
**18∶1n-9**	24.8±1.7a	22.0±1.3a		28.8±0.2a		3.4±0.2a		2.2±0.4a			2.7±0.4a	
**20∶1n-9**	0.7±0.2a	0.7±0.1a		0.8±0.1a		0.2±0.1b		0.2±0.0b			0.0±0.0b	
**20∶1n-11**	0.1±0.1	0.0±0.0		0.0±0.0		0.3±0.2		0.1±0.0			0.0±0.0	
**22∶1n-9**	0.1±0.0	0.0±0.0		0.3±0.0		0.0±0.7		0.0±0.1			0.3±0.1	
**22∶1n-11**	0.0±0.0	0.0±0.0		0.1±0.1		0.1±0.1		0.0±0.0			0.0±0.0	
**24∶1n-9**	1.0±0.1	2.5±0.3a		2.7±0.1a		0.0±0.1		0.1±0.1			0.1±0.1b	
**18∶3n-3**	**1.0±0.2a**	**0.7±0.1**		**1.1±0.4**		**0.2±0.1b**		**0.5±0.1**			**0.1±0.1**	
**18∶4n-3**	**0.1±0.1**	**0.0±0.0**		**0.0±0.0**		**0.2±0.1**		**0.0±0.0**			**0.0±0.0**	
**20∶3n-3**	**1.1±0.1b**	**0.6±0.2b**		**0.8±0.0b**		**34.8±2.3a**		**35.7±3.1a**			**36.5±0.1a**	
**20∶4n-3**	**0.4±0.1**	**0.3±0.1**		**0.5±0.1**		**0.1±0.1**		**0.2±0.1**			**0.3±0.1**	
**20∶5n-3**	**9.4±0.2a**	**5.1±0.9**		**8.3±0.3a**		**6.4±0.8b**		**6.8±0.2**			**7.1±0.1b**	
**22∶3n-3**	**0.0±0.0b**	**0.0±0.0b**		**0.0±0.0b**		**23.4±2.4a**		**24.6±1.9a**			**23.4±1.2a**	
**22∶5n-3**	**3.5±0.3b**	**3.9±0.4b**		**4.8±0.1b**		**8.9±0.8a**		**9.0±0.4a**			**9.5±0.3a**	
**22∶6n-3**	**1.7±0.4b**	**3.0±0.3b**		**3.3±0.1b**		**6.8±0.3a**		**7.0±0.2a**			**7.4±0.7a**	
**18∶2n-6**	1.5±0.4a	1.3±0.5a		1.9±0.1a		0.4±0.1b		0.1±0.1b			0.7±0.1b	
**18∶3n-6**	0.2±0.1	0.3±0.2		0.4±0.1		0.4±0.1		0.4±0.2			0.3±0.1	
**20∶2n-6**	0.6±0.2a	0.7±0.2a		0.8±0.0a		0.0±0.0b		0.0±0.0b			0.1±0.1b	
**20∶3n-6**	0.5±0.2a	1.6±0.3a		1.6±0.0a		0.0±0.0b		0.1±0.1b			0.5±0.2b	
**20∶4n-6**	3.0±0.7a	3.6±0.7a		4.3±0.2a		0.4±0.0b		0.0±0.1b			1.0±0.1b	
**22∶2n-6**	0.1±0.1	0.1±0.0		0.0±0.0		0.0±0.0		0.0±0.0			0.2±0.1	
**22∶4n-6**	0.1±0.0	0.7±0.1a		0.6±0.1a		0.2±0.1		0.0±0.0b			0.1±0.1b	

A 50 µM ALA was supplemented initially to the medium.

Values from the same source (cells, medium or the cells+medium) in the same row with different letters are significantly different (*P*<0.05; ANOVA and Tukey’s post hoc test).

### FA Metabolism Estimation

**Figure 4:**
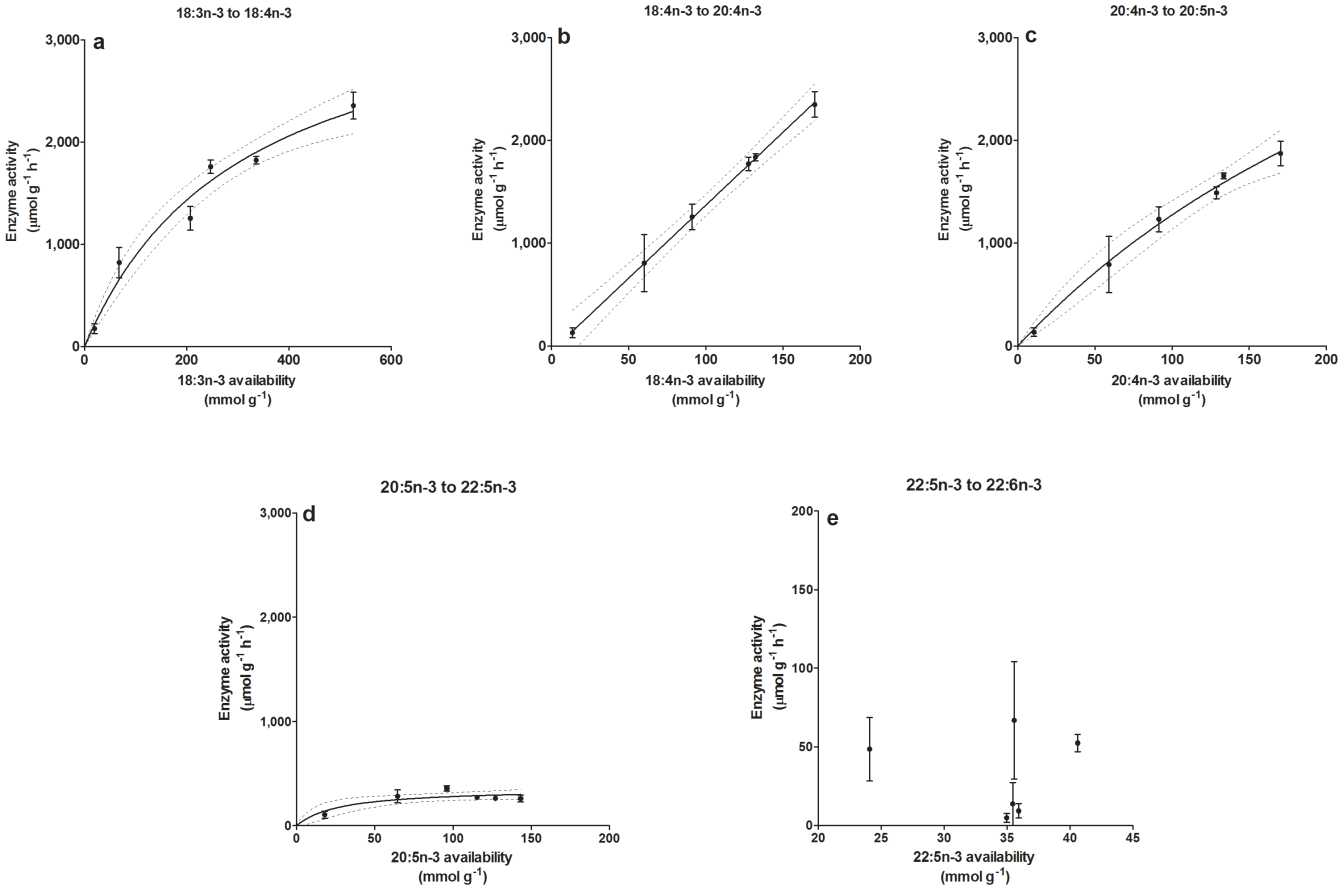
Apparent activity (µmol g^–1^ of cell protein hour of incubation^−1^) of enzymes on different available substrates (mmol g^−1^ of cell protein) in FaO hepatocytes deduced by the fatty acid mass balance. a: Michaelis-Menten nonlinear regression for Fads2 on 18∶3n-3: *Y* = 3654 *X*÷(310+*X*), *R* ^2^ = 0.93, normality of residual = 0.45; b: linear regression for Elovl5 on 18∶4n-3: *Y* = 3.479+14.19 *X*, *R* ^2^ = 0.93, *P* of deviation from zero <0.05; c: Michaelis-Menten nonlinear regression for Fads1 on 20∶4n-3: *Y* = 6052* X÷*(374.6+*X*), *R* ^2^ = 0.89, normality of residuals *P* = 0.26; d: Michaelis-Menten nonlinear regression for Elovl2 and Elovl5 on 20∶5n-3: *Y* = 357.3 *X*÷(27.5+*X*), *R* ^2^ = 0.46, normality of residuals *P* = 0.11; and e: Elovl2, Fads2 and β-oxidation on 22∶5n-3 which is not correlated with the substrate availability. (Note: the *Y* axis of the e subfigure is different from all the others).

Computing the apparent activity of the key enzymes involved in LC-PUFA biosynthesis showed a significant increase in FA Δ-6 desaturase (Fads2) on ALA in response to increasing ALA concentration (Table S7). The apparent Fads2 activity on ALA for the production of SDA was correlated with the availability of ALA (Figure 4a), and it was possible to adequately fit it to a Michaelis-Menten [*Y* = V_max_ *X÷*(Km+*X*)], nonlinear regression:

*Y* = 3654 *X÷*(310+*X*), with *R* ^2^ = 0.93 and (D’Agostino & Pearson omnibus K2 normality of residual *P = *0.45).

Accordingly, the corresponding ALA concentration required to achieve the half-maximum theoretical Fads2 velocity (V_max_ = 3654) during 3 days in FaO cells is 81 µM (Km = 310), and 70%, 80% and 90% of maximum theoretical Fads2 velocity can be achieved by an ALA concentration of 0.189, 0.323 and 0.728 mM, respectively.

The apparent activities of the long-chain FA elongase-5 (Elovl5) acting on SDA for the production of ETA was directly correlated with the availability of the substrate in a significantly positive linear trend (*R* ^2^ = 0.93; slope deviation from zero *P*<0.0001) (Figure 4b). The apparent activity of FA Δ-5 desaturase (Fads1) on ETA for the production of EPA was correlated with the availability of the substrate in a non-linear Michaelis-Menten regression (V_max_ = 6052, Km = 374.6; *R* ^2^ = 0.89 and normality of residual *P = *0.26) (Figure 4c). The apparent activity of Elovl5 and FA elongase-2 (Elovl2) on EPA for the production of DPA was also correlated with the availability of the substrate in a non-linear Michaelis-Menten regression. However, a remarkably lower level of apparent activity (Table S7) as well as low substrate availability was needed to reach a half-maximum enzyme velocity compared with the other enzymes (V_max_ = 357.3, Km = 27.5; *R* ^2^ = 0.46 and normality of residual *P = *0.11) (Figure 4d). The combined apparent activity of Elovl2, Fads2 and β-oxidation (for FA chain shortening) on DPA for the final production of DHA was not correlated with the substrate availability, which varied only from 25 to 40 mmol.g-1 of cell protein (Figure 4e). In a summary, the apparent activities of the key enzymes involved in n-3 LC-PUFA biosynthesis on their substrates are decreasing in the following order: Elovl5>Fads1>Fads2>Elovl2.

## Discussion

In the present study, FaO cells with 50 µM of ALA added recorded a peak of EPA at 3 days, and this was mainly incorporated into the phospholipid fraction of the cells. Accordingly, it is known that compared with monounsaturated FA, PUFA are preferentially esterified by lysophpspholipids acyltransferases in the liver *in vivo* [33], [34] and *in vitro* [35]. Amongst n-3 LC-PUFA, EPA was previously shown to be highly incorporated in HepG2 cell phospholipid compared with DPA and DHA [23], but no further evidence on the incorporation of *de novo* n-3 LC-PUFA into other lipid classes was provided.

A novel observation of this study was that significant amounts of ALA bioconverted products were found to be exported by the cells into the culture medium. It is known that one of the main roles of hepatocytes is to export FA into the bloodstream [36], [37], thus this observation should be expected, despite being rarely considered in previous studies, with only a limited number of exceptions [38], [39]. This can clearly influence the estimation of the dynamics of the bioconversion pathways, as it can be speculated that studies not considering FA composition of the medium, could have actually been underestimating the overall activities of biosynthesis enzymes. Additionally, discarding, or not considering, the medium FA content, can affect the interpretation of results and the understanding of the dynamics of the bioconversion pathways itself, as n-3 LC-PUFA in the medium are reported to have direct feedback on ALA metabolism [11], [22], [40]. Therefore, considering the entire flask FA composition (cells with their culture medium), seems to be necessary for the accurate assessment of the bioconversion dynamics *in vitro*.

The efficiency of ALA bioconversion to EPA and DHA in hepatocytes has been commonly attributed to enzyme affinity, substrate availability and transcriptional factors in experiments assessing FA metabolism in the cells alone [41], [42], but the presence of bioconversion products is also known to have direct effects. In fact, competition between ALA and other n-3 LC-PUFA has been suggested to limit DHA accumulation in hepatocyte membrane *in vitro* [23], [41], and increased availability of n-3 LC-PUFA in medium is known to down regulate the transcription rate of enzymes involved in n-3 LC-PUFA biosynthesis [11], [22], [40]. Therefore, it is suggested that EPA appearing from ALA could be responsible for slowing down the subsequent steps of n-3 LC-PUFA production, with the above mentioned feedback mechanisms. Additionally, compared with other FA, EPA is a robust activator of PPARα [43], a major regulator of genes involved in mitochondrial, peroxisomal and microsomal oxidation [44] which accelerates the rates of oxidation of n-3 LC-PUFA.

Other mammalian hepatocytes have been reported to accumulate high levels of EPA and DPA in cells phospholipid within 1–2 days from supplemented ALA [23], [38], [42], and cell lines from other tissues, such as human colon carcinoma (CaCo-2), had a significant increase in Fads2 enzyme activity at the end of a 3-day experiment [40]. In the present study, the maximal EPA production in FaO cells added with 50 µM of ALA was recorded at 72 h, and therefore 3-days incubation duration was selected for studying the optimal ALA concentration. However, it should be noted that in the following two days, FA composition of cells was not static, and actually, during days 4 and 5 a reduction of EPA and an increase in DPA and DHA levels were apparent, clearly suggesting that a longer time period would have been required for allowing the complete bioconversion of ALA up to DHA. In agreement with this observation, studies tracing ingested labelled-ALA found that DHA take longer time to accumulate in the plasma compared with EPA and DPA [45], [46].

The following dose-response experiment concluded that, as expected, the bioconversion efficiency is also related to the concentration of supplemented ALA. Likewise, it was reported that within a range of 1.8–72 µM ALA supplemented to HepG2 hepatocytes, the accumulation of the phospholipid EPA and DPA, but not DHA, was linearly dependent on the concentration of ALA in the culture medium [23]. Studies on weanling rats have demonstrated a dose-related increase in plasma and liver EPA, DPA and DHA when dietary ALA was 1–3% of dietary energy [10], [47], while a maximal DHA in adult liver was attained with lower levels of dietary ALA (0.26% of dietary energy) [48]. The ALA supplementation used in the current study was in line with that reported in many *in vivo* studies (0–125 µM initial ALA was equal to 0.1–25% of total FA), but also covered a wider range compared with hepatocytes based *in vitro* studies tested previously. A dose-dependent increase in the level of ALA in rat liver was reported to be accompanied by an increase in the level of EPA and DPA [49]. However, increasing dietary ALA did not increase the accumulation of DHA in rat liver [47]. Accordingly, other *in vivo* studies have demonstrated the effect of ALA on increasing EPA accumulation but limiting that of DHA in cell membranes or plasma lipids [13], [50]. The highest ALA concentration tested in the present study (125 µM), was responsible for about 60% of the maximal theoretical activity of Fads2. ALA concentration required for greater Fads2 activity is therefore beyond the currently tested concentrations, but how will this higher concentration affect the possible accumulation of n-3 LC-PUFA still needs to be investigated thoroughly. Within this context, a useful outcome of the current study is that it was shown that if an experimental design is aiming at testing Δ-6 desaturation of ALA under high activity, ALA concentrations of 0.189, 0.323 or 0.728 mM, are needed to achieve 70%, 80% or 90% of its maximum theoretical velocity, respectively.

In the present study, it was clearly shown that, amongst all the bioconversion steps, the fastest and more efficient one was the elongation (Elovl5) of SDA to ETA, followed by the Δ-5 desaturation (Fads1) of ETA to EPA, then the Δ-6 desaturation (Fads2) of ALA to SDA, and eventually the elongation (Elovl2+Elovl5) of EPA to DPA was the slowest recorded. Therefore, a key outcome of the present study was that it was clearly shown that rather than the existence of a single rate-limiting step affecting the overall pathway, a combination of different level of efficiency in each enzymatic step is responsible for the production of n-3 LC-PUFA biosynthesis. It should be noted that the amount of product generated by an enzyme is not only relative to the activity (velocity) of the enzyme itself, but also the time available for this reaction. Accordingly, the elongation of EPA was the slowest recorded step in n-3 LC-PUFA biosynthesis, and because of this limited DPA production, it was not possible to record any specific trend in its further bioconversion towards the final production of DHA. In agreement with this observation, in the first experiment, where cells were incubated for a longer period of time up to 5 days, an increase of DPA was apparent at day 4 and 5, confirming that because of the slow enzyme activity, longer time is required to observe higher level of DPA production, and it is possible that a similar trend would have been followed for DHA production. On the basis of this observation, two important observations should be reported: i) cell culture based studies aiming at assessing DHA production should consider longer incubation time, and ii) further investigations are warranted towards a better understanding of Elovl2, as the possible slowest step in the production of DHA from ALA in hepatocytes. It has been suggested that rat Elovl2, expressed in yeast, controls DHA synthesis from EPA or DPA [51]. Although the regulation of the Elovl2 gene remains to be elucidated [52], some evidence suggests that, compared with that of other FA elongases such as Elovl5, the regulation of Elovl2 expression in rat liver is not influenced by the same environmental or dietary factors [51], [53]. Additionally, and in agreement with the results of the current study, Elovl2 has been reported to convert its substrates at lower rates, compared with Elovl5 in fish [54], chicken [55] and humans [56], [57].

The application of FAMB method in lipid metabolism research has increased in the last few decades as a practical and accurate alternative to costlier analysis [58]–[60]. However, specific variability of an individual sample would need to be taken into consideration, if the method was to be applied to different animals [26], [61]. In culturing established cell lines, maintaining defined equipment and materials of the same high quality guarantees reproducible and reliable results [62], [63]. Therefore, fewer variations are expected to influence FAMB computations when applied on cell culture flasks compared with living animals. The current study has demonstrated FAMB method as a powerful, informative and inexpensive tool for FA metabolism research *in vitro* and to generate detailed description for the kinetics of n-3 LC-PUFA biosynthesis enzymes activities.

## Supporting Information

Table S1 FA changes in FaO hepatocytes at different time-points. A 50 µM ALA was added initially to the culture medium.
(PDF)

Table S2 FA changes in the culture medium of FaO hepatocytes at different time-points. A 50 µM ALA was added initially to the culture medium.
(PDF)

Table S3 FA changes in FaO hepatocytes and their culture medium, combined, at different time-points. A 50 µM ALA was added initially to the culture medium.
(PDF)

Table S4 FA changes after 3 days in FaO hepatocytes cultured with different concentrations of ALA (µM).
(PDF)

Table S5 FA changes after 3 days in FaO hepatocytes culture medium supplemented initially with different concentrations of ALA.
(PDF)

Table S6 FA changes after 3 days in FaO hepatocytes and their culture medium, combined, supplemented initially with different concentrations of ALA.
(PDF)

Table S7 Apparent in vivo activity (µmol of product g^−1^ of protein day^−1^) of key enzymes in the LC-PUFA biosynthetic pathways in FaO hepatocytes culture flask supplemented initially with 50 µM ALA.
(PDF)
