# Supplementary material for: Bioconversion of α-Linolenic Acid into n-3 Long-Chain Polyunsaturated Fatty Acid in Hepatocytes and Ad Hoc Cell Culture Optimisation
Source: PLoS One. 2013 Sep 11;8(9):e73719. doi: 10.1371/journal.pone.0073719 (PMC3770698; doi:10.1371/journal.pone.0073719)
Supplement: Table S1 — FA changes in FaO hepatocytes at different time-points. A 50 µM ALA was added initially to the culture medium. (PDF) [file pone.0073719.s001.pdf]

Table S1: FA changes in FaO hepatocytes at different time-points. A 50 μM ALA was added initially to the culture medium.

**FA %**	**Time (day)**								P^a
	**0**	**0.5**	**1**	**1.5**	**2**	**3**	**4**	**5**	
**12:0**	0.1 ± 0.1	0.0 ± 0.0	0.0 ± 0.0	0.0 ± 0.0	0.0 ± 0.0	0.2 ± 0.1	0.2 ± 0.0	0.2 ± 0.1	0.008
**14:0**	0.8 ± 0.3	0.7 ± 0.5	0.7 ± 0.1	0.5 ± 0.1	1.0 ± 0.3	0.8 ± 0.1	0.9 ± 0.1	0.8 ± 0.0	ns
**16:0**	22.3 ± 4.5	24.2 ± 1.4	20.4 ± 0.7	15.9 ± 0.9	22.1 ± 4.0	17.6 ± 1.3	16.7 ± 0.2	16.2 ± 0.2	0.01
**18:0**	21.1 ± 0.2ab	23.9 ± 1.1a	20.2 ± 3.2ab	20.0 ± 0.3ab	23.1 ± 1.1ab	18.5 ± 0.4ab	17.3 ± 0.5b	16.9 ± 0.2b	0.001
**20:0**	0.6 ± 0.0	0.7 ± 0.1	0.6 ± 0.1	0.7 ± 0.0	0.9 ± 0.1	0.5 ± 0.0	0.6 ± 0.3	0.5 ± 0.1	ns
**22:0**	1.2 ± 0.2a	0.9 ± 0.1a	0.7 ± 0.2a	0.5 ± 0.0ab	0.2 ± 0.2b	0.6 ± 0.0a	0.6 ± 0.0ab	0.6 ± 0.0ab	ns
**14:1n-5**	0.3 ± 0.0a	0.3 ± 0.0a	0.2 ± 0.1ab	0.2 ± 0.0ab	0.4 ± 0.1a	0.2 ± 0.0ab	0.1 ± 0.1bc	0.0 ± 0.0c	0.001
**16:1n-7**	2.2 ± 0.6bcd	1.8 ± 0.7d	2.4 ± 0.2bcd	2.1 ± 0.0bcd	2.0 ± 0.2cd	3.8 ± 0.1ab	4.0 ± 0.2ab	4.3 ± 0.2ab	0.001
**18:1n-7**	4.1 ± 0.3e	3.1 ± 0.5e	5.5 ± 0.2cd	5.1 ± 0.0cd	6.5 ± 0.7bc	6.8 ± 0.4bc	7.9 ± 0.2b	10.5 ± 0.4a	0.001
**18:1n-9**	23.0 ± 0.3a	17.9 ± 0.1b	16.8 ± 2.1b	17.8 ± 0.4b	16.1 ± 1.2b	24.1 ± 0.3a	24.3 ± 1.0a	25.4 ± 0.8a	0.003
**20:1n-9**	1.2 ± 0.2abc	1.2 ± 0.2abc	0.5 ± 0.2c	0.7 ± 0.0bc	1.7 ± 0.4ab	2.3 ± 0.2a	2.1 ± 0.3ab	2.5 ± 0.8a	0.001
**20:1n-11**	3.6 ± 0.6a	3.4 ± 0.4a	2.4 ± 0.9ab	1.9 ± 0.2ab	4.1 ± 2.1a	0.0 ± 0.0b	0.2 ± 0.0b	0.3 ± 0.0b	0.001
**22:1n-9**	0.4 ± 0.4ab	0.0 ± 0.0b	0.9 ± 0.2a	0.7 ± 0.0a	1.3 ± 0.5a	0.6 ± 0.0ab	0.2 ± 0.0ab	0.2 ± 0.1ab	ns
**22:1n-11**	0.7 ± 0.2abc	1.3 ± 0.2a	0.7 ± 0.2abc	0.6 ± 0.0abc	1.1 ± 0.3ab	0.4 ± 0.1abc	0.3 ± 0.0bc	0.2 ± 0.1c	0.001
**24:1n-9**	0.4 ± 0.2ab	0.8 ± 0.2ab	0.7 ± 0.2a	0.6 ± 0.0ab	0.2 ± 0.2b	1.1 ± 0.1ab	1.2 ± 0.1a	1.5 ± 0.1a	0.009
**18:3n-3**	3.3 ± 0.8bc	6.1 ± 0.2ab	9.6 ± 1.6a	7.8 ± 0.4ab	1.8 ± 1.0cd	0.8 ± 0.0cd	0.6 ± 0.1cd	0.4 ± 0.0c	0.001
**18:4n-3**	0.0 ± 0.0c	0.7 ± 0.1a	0.4 ± 0.1a	0.3 ± 0.0ab	0.0 ± 0.0c	0.1 ± 0.1c	0.1 ± 0.1c	0.0 ± 0.0c	0.02
**20:3n-3**	0.0 ± 0.0c	0.6 ± 0.0abc	1.0 ± 0.3ab	2.3 ± 0.1a	1.0 ± 0.6abc	0.6 ± 0.0abc	0.4 ± 0.0bc	0.2 ± 0.1bc	ns
**20:4n-3**	0.6 ± 0.1ab	0.5 ± 0.0ab	1.0 ± 0.3ab	1.7 ± 0.1a	1.1 ± 0.5ab	0.5 ± 0.0ab	0.5 ± 0.0ab	0.2 ± 0.1b	ns
**20:5n-3**	0.3 ± 0.2b	0.1 ± 0.1b	1.6 ± 0.5ab	5.6 ± 0.1a	4.0 ± 2.2ab	6.5 ± 0.3a	5.5 ± 0.2a	4.3 ± 0.0a	0.001
**22:3n-3**	0.0 ± 0.0	0.0 ± 0.0	0.0 ± 0.0	0.0 ± 0.0	0.0 ± 0.0	0.0 ± 0.0	0.0 ± 0.0	0.0 ± 0.0	ns
**22:5n-3**	0.6 ± 0.1c	1.6 ± 0.0bc	2.0 ± 0.1b	2.8 ± 0.1ab	2.9 ± 0.8ab	3.8 ± 0.2a	3.8 ± 0.4a	4.0 ± 0.3a	0.001
**22:6n-3**	1.1 ± 0.2	0.7 ± 0.0	1.5 ± 0.6	1.5 ± 0.1	0.8 ± 0.5	1.6 ± 0.1	1.5 ± 0.0	1.6 ± 0.0	ns
**18:2n-6**	3.4 ± 0.4a	3.4 ± 0.3a	3.2 ± 0.5ab	3.6 ± 0.1a	3.3 ± 0.4ab	2.3 ± 0.2ab	2.4 ± 0.0ab	2.0 ± 0.0b	0.001
**18:3n-6**	2.7 ± 0.2	2.6 ± 0.4	1.5 ± 0.3	2.0 ± 0.1	1.9 ± 0.5	1.7 ± 0.5	1.5 ± 0.5	1.4 ± 0.0	0.01
**20:2n-6**	1.6 ± 0.2ab	1.0 ± 0.0ab	1.1 ± 0.1ab	1.2 ± 0.0ab	0.6 ± 0.3b	1.2 ± 0.1ab	1.9 ± 0.3a	2.0 ± 0.1a	ns
**20:3n-6**	1.6 ± 1.3	0.2 ± 0.2	1.0 ± 0.2	0.7 ± 0.1	0.3 ± 0.3	0.5 ± 0.0	0.5 ± 0.1	0.6 ± 0.0	ns
**20:4n-6**	2.3 ± 0.3	1.8 ± 0.0	3.2 ± 0.7	3.2 ± 0.1	1.6 ± 0.9	2.8 ± 0.1	4.4 ± 1.6	2.9 ± 0.1	ns
**22:2n-6**	0.4 ± 0.2	0.5 ± 0.2	0.3 ± 0.2	0.1 ± 0.1	0.1 ± 0.0	0.1 ± 0.0	0.2 ± 0.2	0.1 ± 0.1	ns
**22:4n-6**	0.0 ± 0.0	0.0 ± 0.0	0.1 ± 0.1	0.0 ± 0.0	0.0 ± 0.0	0.0 ± 0.0	0.1 ± 0.1	0.0 ± 0.0	ns

Values in the same row with different letters are significantly different (P<0.05; ANOVA and Tukey's post hoc test). [a]P value of linear regression reported at 0.05. ns = not significant.
