# Supplementary material for: Bioconversion of α-Linolenic Acid into n-3 Long-Chain Polyunsaturated Fatty Acid in Hepatocytes and Ad Hoc Cell Culture Optimisation
Source: PLoS One. 2013 Sep 11;8(9):e73719. doi: 10.1371/journal.pone.0073719 (PMC3770698; doi:10.1371/journal.pone.0073719)
Supplement: Table S2 — FA changes in the culture medium of FaO hepatocytes at different time-points. A 50 µM ALA was added initially to the culture medium. (PDF) [file pone.0073719.s002.pdf]

Table S2: FA changes in the culture medium of FaO hepatocytes at different time-points. A 50 μM ALA was added initially to the culture medium.

FA %	Time (day)								P^a
	**0**	**0.5**	**1**	**1.5**	**2**	**3**	**4**	**5**	
**12:0**	0.2 ± 0.1	0.1 ± 0.1	0.0 ± 0.0	0.0 ± 0.0	0.1 ± 0.1	0.3 ± 0.1	0.3 ± 0.1	0.1 ± 0.1	ns
**14:0**	1.3 ± 0.1	1.6 ± 0.7	1.0 ± 0.1	1.1 ± 0.0	1.1 ± 0.2	1.3 ± 0.1	1.5 ± 0.1	1.3 ± 0.1	ns
**16:0**	21.9 ± 0.8	29.4 ± 5.7	23.1 ± 0.8	22.8 ± 0.2	24.2 ± 1.0	24.6 ± 0.6	25.6 ± 1.1	24.6 ± 0.1	ns
**18:0**	13.9 ± 0.5	13.2 ± 1.0	17.7 ± 2.8	13.8 ± 0.4	15.6 ± 0.7	14.8 ± 0.2	15.2 ± 0.2	15.5 ± 0.1	ns
**20:0**	0.5 ± 0.1	0.6 ± 0.2	0.6 ± 0.1	0.5 ± 0.0	0.5 ± 0.0	0.5 ± 0.0	0.5 ± 0.0	0.5 ± 0.0	ns
**22:0**	1.1 ± 0.1ab	1.2 ± 0.1a	0.9 ± 0.2ab	1.0 ± 0.0ab	1.0 ± 0.0ab	0.9 ± 0.0ab	0.8 ± 0.0b	0.9 ± 0.0ab	0.003
**14:1n-5**	0.1 ± 0.0a	0.2 ± 0.1a	0.2 ± 0.0a	0.1 ± 0.0a	0.1 ± 0.0a	0.2 ± 0.0a	0.1 ± 0.1b	0.0 ± 0.0b	0.001
**16:1n-7**	2.7 ± 0.1ab	3.2 ± 0.2a	2.5 ± 0.2b	2.9 ± 0.0ab	2.9 ± 0.0ab	2.9 ± 0.1ab	2.8 ± 0.1ab	3.3 ± 0.0a	ns
**18:1n-7**	4.9 ± 0.3b	5.5 ± 0.4ab	5.9 ± 0.1ab	6.5 ± 0.2a	6.3 ± 0.1a	6.4 ± 0.3a	6.3 ± 0.5a	6.7 ± 0.0a	0.001
**18:1n-9**	17.9 ± 0.1	14.2 ± 7.1	19.0 ± 0.3	21.1 ± 0.5	20.4 ± 0.2	21.0 ± 0.3	20.7 ± 0.5	21.7 ± 0.1	ns
**20:1n-9**	0.5 ± 0.1bcd	0.3 ± 0.0d	0.5 ± 0.1bcd	0.3 ± 0.0cd	1.4 ± 0.1ab	1.9 ± 0.6a	2.2 ± 0.7a	1.3 ± 0.1abc	0.001
**20:1n-11**	1.6 ± 0.5ab	1.7 ± 0.2ab	2.1 ± 0.9a	2.1 ± 0.5a	0.1 ± 0.1c	0.2 ± 0.1c	0.2 ± 0.0bc	0.3 ± 0.0bc	0.001
**22:1n-9**	0.3 ± 0.1abc	0.7 ± 0.2a	0.7 ± 0.2a	0.4 ± 0.0ab	0.4 ± 0.0ab	0.1 ± 0.1bc	0.4 ± 0.1ab	0.0 ± 0.0c	0.003
**22:1n-11**	0.2 ± 0.0	0.5 ± 0.1	0.5 ± 0.2	0.4 ± 0.0	0.4 ± 0.1	0.4 ± 0.0	0.3 ± 0.0	0.4 ± 0.1	ns
**24:1n-9**	1.1 ± 0.1	1.0 ± 0.1	0.7 ± 0.4	1.2 ± 0.1	1.4 ± 0.1	1.3 ± 0.0	1.2 ± 0.0	1.6 ± 0.0	ns
**18:3n-3**	14.7 ± 0.6a	7.2 ± 0.2b	7.8 ± 1.8b	4.7 ± 0.3b	2.2 ± 0.2c	0.9 ± 0.1c	1.3 ± 0.1c	0.8 ± 0.0c	0.001
**18:4n-3**	0.3 ± 0.0a	0.2 ± 0.0a	0.3 ± 0.0a	0.1 ± 0.1ab	0.0 ± 0.0b	0.0 ± 0.0b	0.1 ± 0.1b	0.0 ± 0.0b	0.001
**20:3n-3**	0.0 ± 0.0c	0.3 ± 0.1ab	0.6 ± 0.1ab	0.5 ± 0.0ab	0.6 ± 0.2a	0.2 ± 0.0bc	0.2 ± 0.1bc	0.0 ± 0.0c	ns
**20:4n-3**	0.3 ± 0.0b	0.5 ± 0.1ab	0.8 ± 0.2a	0.6 ± 0.1ab	0.6 ± 0.1ab	0.6 ± 0.1ab	0.5 ± 0.1ab	0.5 ± 0.0ab	ns
**20:5n-3**	0.7 ± 0.0b	0.7 ± 0.1b	1.1 ± 0.1ab	1.3 ± 0.1ab	2.6 ± 1.0a	1.8 ± 0.1ab	2.1 ± 0.0a	2.5 ± 0.0a	0.001
**22:3n-3**	0.0 ± 0.0	0.0 ± 0.0	0.0 ± 0.0	0.0 ± 0.0	0.0 ± 0.0	0.0 ± 0.0	0.0 ± 0.0	0.0 ± 0.0	ns
**22:5n-3**	2.3 ± 0.3	2.1 ± 0.3	2.1 ± 0.2	2.5 ± 0.2	2.7 ± 0.4	2.4 ± 0.1	2.2 ± 0.2	2.9 ± 0.1	ns
**22:6n-3**	2.4 ± 0.1	2.1 ± 0.3	1.8 ± 0.7	2.6 ± 0.1	2.8 ± 0.1	2.6 ± 0.1	2.2 ± 0.1	2.5 ± 0.0	ns
**18:2n-6**	4.0 ± 0.1	5.6 ± 1.3	3.6 ± 0.7	4.9 ± 0.1	4.5 ± 0.3	4.7 ± 0.0	4.4 ± 0.1	4.0 ± 0.0	ns
**18:3n-6**	0.6 ± 0.2bc	1.4 ± 0.4abc	1.0 ± 0.3abc	1.0 ± 0.0abc	0.4 ± 0.2c	2.3 ± 0.3a	2.5 ± 0.1a	1.6 ± 0.0ab	0.005
**20:2n-6**	0.5 ± 0.0c	0.5 ± 0.0c	0.7 ± 0.1bc	0.7 ± 0.0bc	0.8 ± 0.1ab	0.8 ± 0.1ab	0.8 ± 0.1ab	1.0 ± 0.0a	0.001
**20:3n-6**	1.3 ± 0.1	1.2 ± 0.1	0.9 ± 0.5	1.4 ± 0.1	1.5 ± 0.1	1.5 ± 0.1	1.2 ± 0.0	1.3 ± 0.1	ns
**20:4n-6**	4.4 ± 0.0	4.3 ± 0.4	3.8 ± 1.0	5.1 ± 0.2	5.3 ± 0.2	5.0 ± 0.2	4.3 ± 0.1	4.7 ± 0.1	ns
**22:2n-6**	0.2 ± 0.1	0.2 ± 0.1	0.2 ± 0.1	0.1 ± 0.0	0.1 ± 0.0	0.1 ± 0.1	0.1 ± 0.1	0.1 ± 0.0	0.02
**22:4n-6**	0.2 ± 0.1	0.2 ± 0.1	0.2 ± 0.1	0.2 ± 0.1	0.2 ± 0.1	0.1 ± 0.1	0.1 ± 0.0	0.1 ± 0.0	0.001

Values in the same row with different letters are significantly different (P<0.05; ANOVA and Tukey's post hoc test). [a]P value of linear regression reported at 0.05. ns = not significant.
