# Supplementary material for: Bioconversion of α-Linolenic Acid into n-3 Long-Chain Polyunsaturated Fatty Acid in Hepatocytes and Ad Hoc Cell Culture Optimisation
Source: PLoS One. 2013 Sep 11;8(9):e73719. doi: 10.1371/journal.pone.0073719 (PMC3770698; doi:10.1371/journal.pone.0073719)
Supplement: Table S3 — FA changes in FaO hepatocytes and their culture medium, combined, at different time-points. A 50 µM ALA was added initially to the culture medium. (PDF) [file pone.0073719.s003.pdf]

Table S3: FA changes in FaO hepatocytes and their culture medium, combined, at different time-points. A 50 µM ALA was added initially to the culture medium.

FA %	Time (day)								P^a
	0	0.5	1	1.5	2	3	4	5	
**12:0**	0.2 ± 0.0ab	0.0 ± 0.0b	0.1 ± 0.0ab	0.0 ± 0.0b	0.0 ± 0.0b	0.2 ± 0.0a	0.1 ± 0.0ab	0.2 ± 0.1a	ns
**14:0**	1.1 ± 0.1	1.0 ± 0.0	0.9 ± 0.1	0.9 ± 0.2	0.7 ± 0.1	0.9 ± 0.1	1.1 ± 0.1	1.0 ± 0.0	ns
**16:0**	20.1 ± 0.4	22.1 ± 1.4	19.8 ± 0.3	21.6 ± 2.0	19.1 ± 2.0	20.4 ± 0.6	20.2 ± 0.6	18.9 ± 0.2	ns
**18:0**	14.1 ± 0.5b	14.1 ± 0.4b	14.0 ± 0.4b	16.4 ± 0.7a	17.9 ± 0.7a	16.0 ± 0.1ab	16.3 ± 0.2ab	15.8 ± 0.1ab	0.01
**20:0**	0.4 ± 0.0c	0.4 ± 0.0bc	0.4 ± 0.0bc	0.5 ± 0.0a	0.6 ± 0.0a	0.5 ± 0.0ab	0.4 ± 0.0c	0.4 ± 0.0c	ns
**22:0**	1.0 ± 0.0	1.0 ± 0.1	1.0 ± 0.0	0.8 ± 0.2	0.9 ± 0.1	0.9 ± 0.0	0.8 ± 0.0	0.8 ± 0.0	0.004
**14:1n-5**	0.1 ± 0.0a	0.1 ± 0.0a	0.1 ± 0.0a	0.3 ± 0.1a	0.1 ± 0.0a	0.0 ± 0.0b	0.0 ± 0.0b	0.0 ± 0.0b	0.001
**16:1n-7**	2.6 ± 0.0c	2.5 ± 0.2c	2.5 ± 0.1c	2.5 ± 0.1c	3.2 ± 0.3bc	3.8 ± 0.1ab	4.2 ± 0.0a	4.0 ± 0.2a	0.001
**18:1n-7**	5.2 ± 0.1e	5.5 ± 0.1de	5.8 ± 0.1cde	5.7 ± 0.2cde	6.4 ± 0.6bcd	6.8 ± 0.2bc	7.6 ± 0.2ab	8.6 ± 0.1a	0.001
**18:1n-9**	18.5 ± 0.0b	19.6 ± 0.9b	19.8 ± 0.1b	20.2 ± 0.3b	21.5 ± 1.5ab	24.2 ± 0.2a	24.7 ± 0.1a	24.8 ± 0.6a	0.001
**20:1n-9**	0.3 ± 0.0d	0.3 ± 0.0d	0.4 ± 0.1cd	1.7 ± 0.7ab	1.8 ± 0.5a	1.7 ± 0.4ab	1.3 ± 0.1abc	1.6 ± 0.4abc	0.001
**20:1n-11**	1.1 ± 0.1ab	1.7 ± 0.2a	0.9 ± 0.0abc	0.4 ± 0.2bcd	0.1 ± 0.1d	0.1 ± 0.1d	0.2 ± 0.0cd	0.2 ± 0.0cd	0.001
**22:1n-9**	0.2 ± 0.0	0.4 ± 0.0	0.3 ± 0.0	0.4 ± 0.1	0.3 ± 0.1	0.4 ± 0.1	0.2 ± 0.0	0.3 ± 0.0	ns
**22:1n-11**	0.2 ± 0.0	0.3 ± 0.0	0.3 ± 0.0	0.5 ± 0.2	0.4 ± 0.1	0.2 ± 0.0	0.2 ± 0.0	0.6 ± 0.3	ns
**24:1n-9**	1.1 ± 0.0	1.0 ± 0.1	1.3 ± 0.1	0.9 ± 0.4	1.3 ± 0.1	1.3 ± 0.0	1.4 ± 0.0	1.5 ± 0.0	ns
**18:3n-3**	15.8 ± 0.7a	11.9 ± 1.1ab	10.5 ± 1.0bc	7.2 ± 0.6c	2.1 ± 0.7d	0.8 ± 0.0d	0.7 ± 0.1d	0.9 ± 0.1d	0.001
**18:4n-3**	0.2 ± 0.0ab	0.2 ± 0.0ab	0.3 ± 0.0a	0.2 ± 0.1abc	0.1 ± 0.0abc	0.0 ± 0.0c	0.0 ± 0.0c	0.0 ± 0.0c	0.001
**20:3n-3**	0.0 ± 0.0d	0.4 ± 0.1c	0.9 ± 0.1bc	1.0 ± 0.3a	1.0 ± 0.2a	0.4 ± 0.0bc	0.4 ± 0.0bc	0.2 ± 0.0c	ns
**20:4n-3**	0.3 ± 0.0d	0.4 ± 0.0cd	0.7 ± 0.0b	1.1 ± 0.1a	0.8 ± 0.1ab	0.5 ± 0.0c	0.4 ± 0.0cd	0.3 ± 0.0cd	ns
**20:5n-3**	0.7 ± 0.0d	0.7 ± 0.1d	1.8 ± 0.1cd	2.6 ± 0.8bc	5.6 ± 0.7a	4.0 ± 0.0ab	4.2 ± 0.1ab	3.9 ± 0.1ab	0.001
**22:3n-3**	0.0 ± 0.0	0.0 ± 0.0	0.0 ± 0.0	0.0 ± 0.0	0.0 ± 0.0	0.0 ± 0.0	0.0 ± 0.0	0.0 ± 0.0	ns
**22:5n-3**	2.1 ± 0.0	2.3 ± 0.1	2.5 ± 0.1	2.2 ± 0.6	3.6 ± 0.4	3.1 ± 0.0	3.5 ± 0.2	3.3 ± 0.4	0.001
**22:6n-3**	2.5 ± 0.0	2.3 ± 0.2	2.8 ± 0.1	2.0 ± 0.7	2.3 ± 0.3	2.3 ± 0.0	2.1 ± 0.0	2.1 ± 0.1	ns
**18:2n-6**	3.8 ± 0.0ab	4.1 ± 0.2a	4.3 ± 0.0a	4.1 ± 0.2a	3.2 ± 0.5ab	3.3 ± 0.2ab	2.8 ± 0.1b	2.8 ± 0.0b	0.001
**18:3n-6**	0.8 ± 0.0ab	0.9 ± 0.1ab	0.9 ± 0.0ab	1.3 ± 0.4a	0.4 ± 0.2b	1.5 ± 0.1a	0.8 ± 0.0ab	0.9 ± 0.1ab	ns
**20:2n-6**	1.0 ± 0.0	0.7 ± 0.1	0.9 ± 0.0	0.6 ± 0.3	1.0 ± 0.2	1.1 ± 0.0	1.3 ± 0.1	1.5 ± 0.0	0.01
**20:3n-6**	1.4 ± 0.0	1.2 ± 0.0	1.4 ± 0.1	0.9 ± 0.5	1.1 ± 0.3	1.1 ± 0.0	1.0 ± 0.0	1.0 ± 0.0	ns
**20:4n-6**	4.7 ± 0.0	4.2 ± 0.3	5.2 ± 0.1	3.9 ± 1.3	4.3 ± 0.7	4.2 ± 0.1	3.9 ± 0.1	4.1 ± 0.1	ns
**22:2n-6**	0.2 ± 0.0a	0.2 ± 0.0a	0.0 ± 0.0b	0.0 ± 0.0b	0.1 ± 0.1ab	0.1 ± 0.1ab	0.0 ± 0.0b	0.1 ± 0.1ab	0.04
**22:4n-6**	0.2 ± 0.0	0.2 ± 0.0	0.3 ± 0.0	0.2 ± 0.1	0.1 ± 0.1	0.2 ± 0.0	0.1 ± 0.1	0.2 ± 0.0	ns

Values in the same row with different letters are significantly different ($P<0.05$; ANOVA and Tukey's post hoc test). [a]*P* value of linear regression reported at 0.05. ns = not significant.
