# Supplementary material for: Bioconversion of α-Linolenic Acid into n-3 Long-Chain Polyunsaturated Fatty Acid in Hepatocytes and Ad Hoc Cell Culture Optimisation
Source: PLoS One. 2013 Sep 11;8(9):e73719. doi: 10.1371/journal.pone.0073719 (PMC3770698; doi:10.1371/journal.pone.0073719)
Supplement: Table S4 — FA changes after 3 days in FaO hepatocytes cultured with different concentrations of ALA (µM). (PDF) [file pone.0073719.s004.pdf]

Table S4: FA changes after 3 days in FaO hepatocytes cultured with different concentrations of ALA (µM).

FA %	Concentration (µM)						P^a
	0	25	50	75	100	125	
**12:0**	0.1 ± 0.0	0.0 ± 0.0	0.0 ± 0.0	0.0 ± 0.0	0.1 ± 0.1	0.0 ± 0.0	ns
**14:0**	1.4 ± 0.2a	0.4 ± 0.1b	0.5 ± 0.1b	0.7 ± 0.1b	0.9 ± 0.2ab	0.4 ± 0.1b	ns
**16:0**	38.7 ± 2.2a	32.2 ± 3.5ab	22.5 ± 2.6bc	23.1 ± 0.6bc	17.8 ± 2.7c	16.0 ± 1.6c	0.003
**18:0**	16.8 ± 0.3c	18.2 ± 0.4bc	21.1 ± 0.8ab	19.9 ± 0.4abc	23.0 ± 0.9a	23.5 ± 1.3a	0.001
**20:0**	0.7 ± 0.0a	0.9 ± 0.1a	0.7 ± 0.0a	0.6 ± 0.1ab	0.2 ± 0.1b	0.3 ± 0.0b	0.002
**22:0**	0.7 ± 0.1	0.8 ± 0.1	0.7 ± 0.0	0.9 ± 0.1	0.7 ± 0.1	0.7 ± 0.0	ns
**14:1n-5**	0.0 ± 0.0b	0.0 ± 0.0b	0.0 ± 0.0b	0.0 ± 0.0b	0.3 ± 0.1a	0.0 ± 0.0b	ns
**16:1n-7**	3.6 ± 0.4	4.5 ± 1.2	3.1 ± 0.3	3.1 ± 0.3	2.9 ± 0.1	2.5 ± 0.2	0.01
**18:1n-7**	6.7 ± 0.5	6.4 ± 0.4	5.5 ± 1.9	5.3 ± 0.4	5.7 ± 0.2	2.2 ± 2.0	0.02
**18:1n-9**	19.2 ± 1.4b	20.2 ± 0.2ab	24.8 ± 1.7a	23.0 ± 0.3ab	20.9 ± 0.4ab	22.9 ± 1.3ab	ns
**20:1n-9**	0.6 ± 0.1a	0.6 ± 0.0a	0.6 ± 0.0a	0.1 ± 0.1b	0.4 ± 0.0a	0.4 ± 0.0a	ns
**20:1n-11**	0.0 ± 0.0b	0.0 ± 0.0b	0.0 ± 0.0b	0.0 ± 0.0b	0.0 ± 0.0b	0.3 ± 0.1a	0.003
**22:1n-9**	0.2 ± 0.0	0.8 ± 0.6	0.7 ± 0.3	1.4 ± 0.7	1.4 ± 0.0	1.3 ± 0.2	0.03
**22:1n-11**	0.0 ± 0.0	0.0 ± 0.0	0.0 ± 0.0	0.7 ± 0.4	0.0 ± 0.0	0.4 ± 0.2	ns
**24:1n-9**	0.9 ± 0.1b	1.4 ± 0.2ab	1.3 ± 0.1ab	1.3 ± 0.1ab	1.6 ± 0.1a	1.7 ± 0.1a	0.001
**18:3n-3**	0.1 ± 0.0d	0.2 ± 0.1d	0.5 ± 0.1c	0.7 ± 0.0c	1.3 ± 0.1b	1.9 ± 0.1a	0.001
**18:4n-3**	0.2 ± 0.0	0.1 ± 0.1	0.0 ± 0.0	0.2 ± 0.1	0.0 ± 0.0	0.0 ± 0.0	0.01
**20:3n-3**	0.7 ± 0.0	1.2 ± 0.4	0.6 ± 0.1	0.5 ± 0.1	0.5 ± 0.0	0.8 ± 0.1	ns
**20:4n-3**	0.0 ± 0.0b	0.0 ± 0.0b	0.0 ± 0.0b	1.8 ± 0.2a	2.0 ± 0.3a	2.3 ± 0.2a	0.001
**20:5n-3**	0.5 ± 0.0d	2.7 ± 0.2c	5.7 ± 0.6b	6.5 ± 0.5b	10.3 ± 0.6a	11.8 ± 0.7a	0.002
**22:3n-3**	0.0 ± 0.0	0.0 ± 0.0	0.0 ± 0.0	0.0 ± 0.0	0.0 ± 0.0	0.0 ± 0.0	ns
**22:5n-3**	0.7 ± 0.1c	1.7 ± 0.1b	3.0 ± 0.2a	2.4 ± 0.2ab	2.9 ± 0.2a	3.0 ± 0.2a	0.001
**22:6n-3**	0.8 ± 0.1b	1.0 ± 0.1b	1.4 ± 0.1a	1.0 ± 0.1b	1.3 ± 0.1a	1.3 ± 0.1a	0.001
**18:2n-6**	1.4 ± 0.1b	1.5 ± 0.1b	1.7 ± 0.1ab	1.8 ± 0.2a	1.7 ± 0.1ab	2.0 ± 0.2a	0.009
**18:3n-6**	1.5 ± 0.1a	1.0 ± 0.2ab	1.4 ± 0.1a	0.8 ± 0.1b	0.2 ± 0.2c	0.0 ± 0.0c	0.005
**20:2n-6**	1.5 ± 0.1a	1.0 ± 0.1b	1.0 ± 0.1b	0.5 ± 0.1c	0.5 ± 0.0c	0.4 ± 0.0c	0.001
**20:3n-6**	0.3 ± 0.0b	0.3 ± 0.0ab	0.4 ± 0.0a	0.4 ± 0.0ab	0.4 ± 0.0ab	0.4 ± 0.0a	0.002
**20:4n-6**	1.7 ± 0.1b	1.9 ± 0.1ab	2.6 ± 0.1a	2.0 ± 0.2ab	2.6 ± 0.2a	2.6 ± 0.1a	0.004
**22:2n-6**	0.2 ± 0.0a	0.0 ± 0.0b	0.0 ± 0.0b	0.0 ± 0.0b	0.0 ± 0.0b	0.0 ± 0.0b	0.003
**22:4n-6**	0.7 ± 0.1	0.8 ± 0.3	0.2 ± 0.2	1.4 ± 0.4	0.6 ± 0.3	0.8 ± 0.2	ns

Values in the same row with different letters are significantly different (P<0.05; ANOVA and Tukey's post hoc test). [a]P value of linear regression reported at 0.05. ns = not significant
