# Supplementary material for: Bioconversion of α-Linolenic Acid into n-3 Long-Chain Polyunsaturated Fatty Acid in Hepatocytes and Ad Hoc Cell Culture Optimisation
Source: PLoS One. 2013 Sep 11;8(9):e73719. doi: 10.1371/journal.pone.0073719 (PMC3770698; doi:10.1371/journal.pone.0073719)
Supplement: Table S5 — FA changes after 3 days in FaO hepatocytes culture medium supplemented initially with different concentrations of ALA. (PDF) [file pone.0073719.s005.pdf]

Table S5: FA changes after 3 days in FaO hepatocytes culture medium supplemented initially with different concentrations of ALA.

FA %	Concentration (µM)						P^a
	0	25	50	75	100	125	
**12:0**	0.0 ± 0.0	0.0 ± 0.0	0.0 ± 0.0	0.0 ± 0.0	0.0 ± 0.0	0.0 ± 0.0	ns
**14:0**	1.1 ± 0.1	1.0 ± 0.1	0.8 ± 0.2	0.8 ± 0.2	0.9 ± 0.3	1.4 ± 0.2	ns
**16:0**	38.4 ± 2.6a	37.8 ± 6.1ab	27.0 ± 0.7abc	24.7 ± 2.2abc	24.2 ± 1.5bc	22.9 ± 0.4c	0.001
**18:0**	17.3 ± 0.3ab	16.3 ± 0.2b	18.6 ± 0.3a	18.6 ± 0.6a	19.0 ± 0.4a	17.7 ± 0.5ab	ns
**20:0**	0.9 ± 0.1a	0.9 ± 0.2ab	0.5 ± 0.1abc	0.5 ± 0.1abc	0.4 ± 0.0bc	0.3 ± 0.0c	0.001
**22:0**	0.8 ± 0.1	0.8 ± 0.1	0.6 ± 0.0	0.7 ± 0.0	0.7 ± 0.0	0.7 ± 0.1	ns
**14:1n-5**	0.0 ± 0.0	0.0 ± 0.0	0.2 ± 0.2	0.1 ± 0.1	0.1 ± 0.1	0.0 ± 0.0	ns
**16:1n-7**	5.2 ± 0.4a	3.4 ± 0.2b	3.4 ± 0.2b	2.5 ± 0.1b	2.7 ± 0.1b	3.0 ± 0.3b	0.001
**18:1n-7**	3.9 ± 0.5ab	4.4 ± 0.9a	0.9 ± 0.1bc	5.0 ± 0.3a	0.4 ± 0.1c	1.9 ± 1.5abc	0.03
**18:1n-9**	15.2 ± 1.8b	16.6 ± 2.7ab	23.3 ± 0.3a	19.8 ± 0.2ab	20.1 ± 0.4ab	19.3 ± 0.9ab	ns
**20:1n-9**	0.4 ± 0.1	0.4 ± 0.1	0.4 ± 0.0	0.2 ± 0.2	0.2 ± 0.2	0.3 ± 0.0	ns
**20:1n-11**	0.0 ± 0.0b	0.0 ± 0.0b	0.0 ± 0.0b	0.0 ± 0.0b	0.1 ± 0.1ab	0.3 ± 0.0a	0.001
**22:1n-9**	0.4 ± 0.2b	0.7 ± 0.4ab	0.0 ± 0.0c	1.4 ± 0.1a	1.4 ± 0.1a	1.0 ± 0.1ab	0.03
**22:1n-11**	0.0 ± 0.0c	0.0 ± 0.0c	0.0 ± 0.0c	0.6 ± 0.0a	0.7 ± 0.0a	0.5 ± 0.0b	0.001
**24:1n-9**	1.3 ± 0.1bc	1.3 ± 0.1bc	1.0 ± 0.0c	1.9 ± 0.1a	1.8 ± 0.1ab	1.6 ± 0.1ab	0.01
**18:3n-3**	0.3 ± 0.0e	0.8 ± 0.2de	1.3 ± 0.2cd	1.6 ± 0.1bc	2.6 ± 0.1b	4.6 ± 0.3a	0.001
**18:4n-3**	0.4 ± 0.0a	0.2 ± 0.1ab	0.0 ± 0.0b	0.2 ± 0.1ab	0.0 ± 0.0b	0.0 ± 0.0b	0.001
**20:3n-3**	1.4 ± 0.2a	0.9 ± 0.1ab	0.7 ± 0.1ab	0.6 ± 0.1b	0.8 ± 0.0ab	0.9 ± 0.2ab	ns
**20:4n-3**	0.0 ± 0.0b	0.0 ± 0.0b	0.0 ± 0.0b	2.9 ± 0.2a	2.9 ± 0.2a	3.1 ± 0.6a	0.001
**20:5n-3**	0.8 ± 0.1c	1.4 ± 0.2c	3.6 ± 0.2b	4.0 ± 0.4ab	4.9 ± 0.2a	5.2 ± 0.1a	0.001
**22:3n-3**	0.0 ± 0.0	0.0 ± 0.0	0.0 ± 0.0	0.0 ± 0.0	0.0 ± 0.0	0.0 ± 0.0	ns
**22:5n-3**	1.1 ± 0.1b	1.3 ± 0.3b	2.4 ± 0.0a	2.0 ± 0.2a	2.3 ± 0.1a	2.1 ± 0.1a	0.001
**22:6n-3**	1.4 ± 0.2b	1.7 ± 0.4b	2.6 ± 0.0a	2.1 ± 0.2ab	2.5 ± 0.1a	2.3 ± 0.2ab	0.004
**18:2n-6**	2.7 ± 0.3b	3.1 ± 0.4b	4.1 ± 0.2a	3.5 ± 0.2ab	4.2 ± 0.1a	4.0 ± 0.3a	0.003
**18:3n-6**	2.2 ± 0.2a	1.8 ± 0.1a	1.3 ± 0.3ab	0.5 ± 0.2bc	0.0 ± 0.0c	0.0 ± 0.0c	0.001
**20:2n-6**	0.7 ± 0.0	0.6 ± 0.2	0.7 ± 0.0	0.5 ± 0.0	0.6 ± 0.0	0.5 ± 0.0	ns
**20:3n-6**	0.7 ± 0.1b	0.8 ± 0.2ab	1.2 ± 0.0a	1.0 ± 0.1ab	1.2 ± 0.1a	1.1 ± 0.1ab	0.003
**20:4n-6**	2.5 ± 0.4a	3.2 ± 0.7ab	5.0 ± 0.1a	4.0 ± 0.4ab	4.9 ± 0.1a	4.6 ± 0.4a	0.003
**22:2n-6**	0.0 ± 0.0	0.1 ± 0.1	0.0 ± 0.0	0.0 ± 0.0	0.0 ± 0.0	0.0 ± 0.0	ns
**22:4n-6**	1.0 ± 0.2a	0.7 ± 0.1ab	0.3 ± 0.2b	0.4 ± 0.0ab	0.5 ± 0.0ab	0.6 ± 0.1ab	ns

Values in the same row with different letters are significantly different (P<0.05; ANOVA and Tukey's post hoc test). [a]P value of linear regression reported at 0.05. ns = not significant
