# Supplementary material for: Bioconversion of α-Linolenic Acid into n-3 Long-Chain Polyunsaturated Fatty Acid in Hepatocytes and Ad Hoc Cell Culture Optimisation
Source: PLoS One. 2013 Sep 11;8(9):e73719. doi: 10.1371/journal.pone.0073719 (PMC3770698; doi:10.1371/journal.pone.0073719)
Supplement: Table S6 — FA changes after 3 days in FaO hepatocytes and their culture medium, combined, supplemented initially with different concentrations of ALA. (PDF) [file pone.0073719.s006.pdf]

Table S6: FA changes after 3 days in FaO hepatocytes and their culture medium, combined, supplemented initially with different concentrations of ALA.

FA %	Concentration (µM)						P^a
	0	25	50	75	100	125	
**12:0**	0.0 ± 0.0	0.0 ± 0.0	0.0 ± 0.0	0.0 ± 0.0	0.0 ± 0.0	0.0 ± 0.0	ns
**14:0**	0.9 ± 0.1	0.6 ± 0.0	0.7 ± 0.1	0.8 ± 0.1	0.5 ± 0.1	0.8 ± 0.1	ns
**16:0**	28.4 ± 2.7	27.5 ± 6.9	19.1 ± 1.5	19.8 ± 0.6	16.1 ± 0.4	17.8 ± 1.1	0.002
**18:0**	15.9 ± 0.4c	16.0 ± 0.4c	18.4 ± 0.5b	20.2 ± 0.6ab	20.9 ± 0.1a	20.7 ± 0.2a	0.001
**20:0**	0.8 ± 0.1a	0.6 ± 0.1ab	0.4 ± 0.1ab	0.3 ± 0.0b	0.3 ± 0.0b	0.3 ± 0.0b	0.001
**22:0**	1.0 ± 0.0a	0.7 ± 0.1b	0.8 ± 0.0ab	0.8 ± 0.0b	0.7 ± 0.0b	0.7 ± 0.0b	0.002
**14:1n-5**	0.0 ± 0.0	0.0 ± 0.0	0.1 ± 0.1	0.2 ± 0.2	0.0 ± 0.0	0.1 ± 0.1	ns
**16:1n-7**	4.1 ± 0.2a	3.3 ± 0.4ab	3.5 ± 0.1ab	3.0 ± 0.2ab	2.8 ± 0.1b	2.8 ± 0.1b	0.001
**18:1n-7**	8.1 ± 0.5a	8.3 ± 1.1a	7.8 ± 0.2ab	6.2 ± 0.2ab	6.3 ± 0.1ab	5.5 ± 0.1b	0.001
**18:1n-9**	23.7 ± 1.2ab	24.4 ± 3.0ab	26.2 ± 0.4a	22.7 ± 0.2ab	22.4 ± 0.2ab	19.3 ± 0.3b	0.02
**20:1n-9**	0.7 ± 0.0a	0.7 ± 0.1a	0.6 ± 0.0ab	0.5 ± 0.0bc	0.4 ± 0.0c	0.3 ± 0.0c	0.001
**20:1n-11**	0.0 ± 0.0	0.0 ± 0.0	0.0 ± 0.0	0.0 ± 0.0	0.1 ± 0.1	0.1 ± 0.1	ns
**22:1n-9**	0.6 ± 0.2	0.4 ± 0.0	0.5 ± 0.1	0.5 ± 0.3	0.0 ± 0.0	0.7 ± 0.3	ns
**22:1n-11**	0.0 ± 0.0b	0.0 ± 0.0b	0.0 ± 0.0b	0.4 ± 0.0a	0.3 ± 0.0a	0.3 ± 0.1a	0.001
**24:1n-9**	1.8 ± 0.1	1.3 ± 0.2	1.6 ± 0.2	1.8 ± 0.1	1.5 ± 0.0	1.4 ± 0.1	ns
**18:3n-3**	0.2 ± 0.1e	0.4 ± 0.1e	0.7 ± 0.1d	1.2 ± 0.0c	2.3 ± 0.1b	3.8 ± 0.4a	0.001
**18:4n-3**	0.2 ± 0.0a	0.1 ± 0.0b	0.1 ± 0.0b	0.1 ± 0.0b	0.1 ± 0.0b	0.1 ± 0.0b	ns
**20:3n-3**	0.5 ± 0.0b	0.3 ± 0.1b	0.3 ± 0.0b	0.4 ± 0.0b	0.5 ± 0.0ab	0.9 ± 0.0a	0.01
**20:4n-3**	0.0 ± 0.0d	0.1 ± 0.0c	0.2 ± 0.0c	1.5 ± 0.0b	1.2 ± 0.0b	2.5 ± 0.1a	0.001
**20:5n-3**	0.6 ± 0.0d	2.7 ± 0.4c	5.3 ± 0.7b	6.8 ± 0.3ab	9.1 ± 0.1a	8.7 ± 0.3a	0.001
**22:3n-3**	0.0 ± 0.0	0.0 ± 0.0	0.0 ± 0.0	0.0 ± 0.0	0.0 ± 0.0	0.0 ± 0.0	0.001
**22:5n-3**	1.3 ± 0.1c	2.1 ± 0.3b	3.0 ± 0.2a	2.7 ± 0.1ab	3.2 ± 0.1a	2.6 ± 0.1ab	0.001
**22:6n-3**	1.6 ± 0.1	1.7 ± 0.3	2.0 ± 0.1	1.8 ± 0.1	2.1 ± 0.0	1.8 ± 0.1	ns
**18:2n-6**	2.2 ± 0.1	2.3 ± 0.3	2.5 ± 0.1	2.6 ± 0.1	2.7 ± 0.0	2.6 ± 0.1	0.003
**18:3n-6**	1.2 ± 0.1a	0.7 ± 0.1ab	0.4 ± 0.0b	0.7 ± 0.3ab	0.0 ± 0.0c	0.0 ± 0.0c	0.001
**20:2n-6**	1.8 ± 0.1a	1.3 ± 0.1b	0.9 ± 0.0bc	0.6 ± 0.1d	0.7 ± 0.0cd	0.5 ± 0.1d	0.001
**20:3n-6**	0.7 ± 0.0	0.7 ± 0.1	0.8 ± 0.1	0.8 ± 0.1	0.9 ± 0.0	0.8 ± 0.0	ns
**20:4n-6**	3.2 ± 0.2	3.3 ± 0.5	3.8 ± 0.3	3.5 ± 0.2	4.3 ± 0.1	3.7 ± 0.2	0.03
**22:2n-6**	0.1 ± 0.0	0.0 ± 0.0	0.0 ± 0.0	0.0 ± 0.0	0.0 ± 0.0	0.0 ± 0.0	0.01
**22:4n-6**	0.3 ± 0.0b	0.4 ± 0.1b	0.3 ± 0.1b	0.3 ± 0.0b	0.3 ± 0.0b	0.7 ± 0.1a	0.02

Values in the same row with different letters are significantly different ($P<0.05$; ANOVA and Tukey's post hoc test). [a]*P* value of linear regression reported at 0.05. ns = not significant.
