# Supplementary material for: Bioconversion of α-Linolenic Acid into n-3 Long-Chain Polyunsaturated Fatty Acid in Hepatocytes and Ad Hoc Cell Culture Optimisation
Source: PLoS One. 2013 Sep 11;8(9):e73719. doi: 10.1371/journal.pone.0073719 (PMC3770698; doi:10.1371/journal.pone.0073719)
Supplement: Table S7 — Apparent in vivo activity (µmol of product g−1 of protein day−1) of key enzymes in the LC-PUFA biosynthetic pathways in FaO hepatocytes culture flask supplemented initially with 50 µM ALA. (PDF) [file pone.0073719.s007.pdf]

Table S7: Apparent *in vivo* activity (µmol of product g^{-1} of protein h^{-1}) of key enzymes in the LC-PUFA biosynthetic pathways in FaO hepatocytes culture flask supplemented initially with 50 µM ALA

Activity	Concentration (µM)																	
	0			25			50			75			100			125		
Δ-6 desaturase																		
**18:2n-6 to 18:3n-6**	0.0	±	0.0	0.2		0.1	0.1	±	0.1	0.0	±	0.0	0.1	±	0.1	0.0	±	0.0
**24:5n-6 to 24:6n-6**	0.0	±	0.0	0.0	±	0.0	0.0	±	0.0	0.0	±	0.0	0.0	±	0.0	0.0	±	0.0
**18:3n-3 to 18:4n-3**	173.8	±	27.6e	821.0	±	18.9d	1253.6	±	51.9c	1757.8	±	65.3b	1822.5	±	35.8b	2354.3	±	31.7a
**24:5n-3 to 24:6n-3**	48.4	±	6.2	66.8	±	8.5	52.4	±	5.5	9.2	±	0.6	4.8	±	0.9	13.6	±	3.6
Δ-5 desaturase																		
**20:3n-6 to 20:4n-6**	138.2	±	11.8a	159.8	±	16.4a	108.4	±	16.9a	32.6	±	5.5d	61.3	±	4.7c	75.6	±	3.9b
**20:4n-3 to 20:5n-3**	136.0	±	19.6e	792.0	±	22.1d	1232.4	±	31.6c	1490.1	±	40.4bc	1654.2	±	29.0ab	1873.2	±	30.9a
Elongase 5																		
**18:4n3 to 20:4n-3**	129.2	±	29.1e	806.9	±	57.3d	1256.5	±	124.2c	1772.2	±	64.5b	1836.7	±	43.8b	2349.1	±	125.4a
**18:3n-6 to 20:3n-6**	128.8		34.2	162.8		28.3	94.8		22.1	11.8		3.4	24.6		6.5	46.8		11.9
Elongase 2 and 5																		
**20:5n-3 to 22:5n-3**	106.1	±	25.2c	283.2	±	32.4b	358.6	±	25.2a	271.6	±	17.1b	262.9	±	19.1b	260.5	±	32.1b
**20:4n-6 to 22:4n-6**	0.0	±	0.0	0.0	±	0.0	0.0	±	0.0	0.0	±	0.0	0.0	±	0.0	0.0	±	0.0
Elongase 2																		
**22:5n-3 to 24:5n-3**	48.4	±	10.2a	66.8	±	16.5a	52.4	±	5.5a	9.2	±	2.6b	4.8	±	0.9c	13.6	±	1.9b
**22:4n-6 to 24:4n-6**	0.0	±	0.0	0.0	±	0.0	0.0	±	0.0	0.0	±	0.0	0.0	±	0.0	0.0	±	0.0
Peroxisomal chain shortening																		
**24:5n-6 to 22:5n-6**	0.0	±	0.0	0.0	±	0.0	0.0	±	0.0	0.0	±	0.0	0.0	±	0.0	0.0	±	0.0
**24:6n-3 to 22:6n-3**	48.4	±	2.2c	66.8	±	5.5a	52.4	±	5.5b	9.2	±	0.6e	4.8	±	0.9f	13.6	±	1.6d
E+ Δ-6+CS[b]																		
**22:5n-3 to 22:6n-3**	145.3	±	20.2a	200.3	±	56.5a	157.3	±	5.5a	27.6	±	4.6c	14.5	±	2.9c	40.9	±	13.6b

Values in the same row with different letters are significantly different (*P*<0.05; ANOVA and Tukey's post hoc test). [a]*P* value of linear regression reported at 0.05. ns = not significant. [b] E+ Δ-6+CS = elongase (22:5n-3 to 24:5n-3) + Δ-6 desaturase (24:5n-3 to 24:6n-3) + chain shortening (24:6n-3 to 22:6n-3).
